# Toxicological Assessment of Flavor Ingredients in E-Vapor Products

**DOI:** 10.3389/ftox.2022.878976

**Published:** 2022-04-20

**Authors:** Davide Sciuscio, Florian Calvino-Martin, Ashutosh Kumar, Timothy B. Langston, Elyette Martin, Diego Marescotti, Carole Mathis, Julia Hoeng, Manuel C. Peitsch, Donna C. Smith, Maria Gogova, Patrick Vanscheeuwijck, Kyeonghee M. Lee

**Affiliations:** ^1^ PMI R&D, Philip Morris Products S.A., Neuchâtel, Switzerland; ^2^ Altria Client Services LLC, Richmond, VA, United States

**Keywords:** reduced risk products, flavor ingredients, structural grouping, *in silico* toxicology, E-vapor products, ENDS

## Abstract

Many flavor ingredients are often used in potentially reduced-risk tobacco products (such as e-vapor products). Although most are “generally recognized as safe (GRAS)” when used in food, there is limited information available on their long-term health effects when delivered by inhalation. While obtaining route-of-exposure-specific toxicological data on flavor ingredients is critical to product evaluation, the large number of individual flavor ingredients available and their potential combinations render classical toxicological assessment approaches impractical, as they may require years of preclinical investigations and thousands of laboratory animals. Therefore, we propose a pragmatic approach in which flavor ingredients are initially assigned to groups of structurally related compounds (Flavor Groups), from which flavor group representatives (FGR) are then selected and tested individually and as a mixture *in vitro* and *in vivo*. The premise is that structurally related compounds would have comparable metabolic and biological activity and that the data generated using FGRs could support the toxicological assessment of other structurally related flavor ingredients of their respective Flavor Groups. This approach is explained in a step-wise manner and exemplified by a case study, along with its strengths, limitations as well as recommendations for further confirmatory testing. Once completed, this FGR approach could significantly reduce the time and resources required for filling the data gap in understanding the health risks of many flavor ingredients while also minimizing the need for laboratory animals.

## 1 Introduction

### 1.1 Role of Flavor Ingredients in Reduced-Risk Products

While the prevalence of cigarette smoking is declining in many developed countries (Global Burden of Diseases, 2017), the actual number of adult smokers is still significant. For instance, there are over 34 million current smokers in the United States (US) (NHIS, 2018; U.S. Centers for Disease Controland Prevention, 2019) and cigarette smoking remains the leading cause of major preventable diseases, morbidity, and mortality. While public health strategies for reducing smoking-related harm have focused on preventing smoking initiation and promoting smoking cessation (U.S. Surgeon General, 2010; U.S. Surgeon General, 2014; Royal College of Physicians, 2016), many smokers are either unwilling or unable to quit and the long-term cessation success rate remains very low with most quit attempts often failing within the first year (Hughes et al., 2004; U.S. Surgeon General, 2010; Medicine, 2012; Royal College of Physicians, 2016). As former US Food and Drug Administration (FDA) Commissioner, Scott Gottlieb, pointed out, “we must recognize the potential for innovative, less harmful products that can efficiently deliver satisfying levels of nicotine to adults who want them (U.S. Food and Drug Administration, 2018). In the United Kingdom, the use of reduced-risk tobacco products (RRPs), in particular e-vapor products, is viewed as a promising tobacco harm reduction (THR) strategy. An expert review published by the Public Health England (PHE) stated that “vaping poses only a small fraction of the risks of smoking and switching completely from smoking to vaping conveys substantial health benefits (McNeill et al., 2018). At the same time, the long-term health risks of e-vapor and flavor ingredient exposures are unknown and further investigations in long-term adverse effects are necessary (National Academies of Sciences, 2018). Currently a variety of potential RRPs are available around the world, including smokeless oral tobacco products such as snus, “tobacco-free” oral nicotine products, heated tobacco products (e.g., IQOS®, Phillip Morris International), and electronic nicotine delivery systems (ENDS) or e-vapor products. While promising, the long-term success of complete switching from cigarettes to RRPs is unknown (Polosa et al., 2013; Farsalinos et al., 2018). If smokers do not accept these products as sustainable alternatives to cigarettes regardless of how low their toxicity profile is, they will not switch, and cigarette smoking cessation will not occur. Among RRP candidates, e-vapor products deliver nicotine-containing aerosols via inhalation, potentially enabling rapid nicotine uptake and a sensorial experience overall similar to smoking (Voos et al., 2020). As postulated in Abrams et al., e-vapor products are in a unique position to achieve THR for adult smokers in terms of (a) (reduced) harmfulness, (b) appeal, and (c) satisfaction (Abrams et al., 2018). The Institute of Medicine similarly describes the importance of product appeal to a smoker seeking RRPs to switch: “Ideally, a reduced risk tobacco product would be sufficiently reinforcing so as to attract smokers away from conventional cigarettes but not encourage the widespread dependent use of the product by individuals who were previously nonusers or who would have quit smoking.” (Medicine, 2012). The “sweet spot” on the THR model is where RRPs are substantially lower than cigarettes in toxicity/harmfulness, relatively high in appeal to current tobacco users but not adversely impacting initiation by non-smokers including youths (Smith et al., 2016; Abrams et al., 2018; Warner, 2019).

**FIGURE 1:**
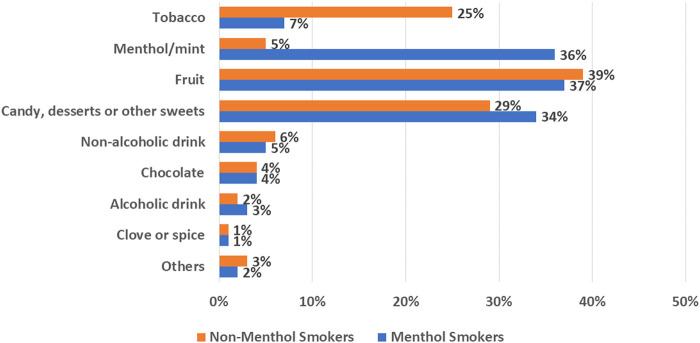
Path wave 4 adult dual cigarette and E-vapor product consumers, percent reporting E-vapor product flavor use in past 30 Days.

Among product- -related factors that would affect the liking and acceptability of RRPs, flavor ingredients as well as nicotine contribute to taste and sensory experiences that may enhance satisfaction and switching away from cigarettes. The US Population Assessment of Tobacco and Health (PATH) survey data suggest the potential role of flavor ingredients to the e-vapor product user experience that adult e-vapor users engage with a wide variety of e-liquid flavor ingredients, with preferences for non-tobacco flavor ingredients (e.g., fruit, mint, or menthol flavors; Figure 1). Several studies show that e-vapor product users frequently report first using or buying a flavored e-vapor product (Harrell et al., 2017; Russell et al., 2018; Landry et al., 2019; Villanti et al., 2019; Vu et al., 2019) and having diverse flavor options was critical to aid continued e-vapor product (only) use over smoking (Chen et al., 2018; Friedman and Xu, 2020).

### 1.2 Gap in Inhalation Toxicity Profiles of Flavor Ingredients in E-Vapor Products

While RRPs, such as e-vapor products, are designed to be substantially less toxic than cigarettes (e.g., lack of tobacco combustion), no tobacco product (including RRPs)is risk-free. For e-vapor products, the long-term health risks of their aerosols, which contain humectants and flavor ingredients, have not yet been assessed and it will take decades before any potential long term health impacts associated with chronic e-vapor use are fully understood. The National Academies of Sciences, Engineering, and Medicine confirmed this notion, suggesting “that e-cigarettes are not without physiological activity in humans” and that further investigations are needed (National Academies of Sciences, 2018). Many non-nicotine ingredients used in e-vapor products [e.g., flavor ingredients and humectants such as propylene glycol [PG] and vegetable glycerol (VG)] are GRAS for oral consumption in food, but in general have insufficient toxicity data available when exposure occurs via inhalation (Committee on Toxicity of Chemicals in Food C.P.a.t.E.C, 2016). While safety profiles via oral exposure provide some assurance in terms of systemic toxicity, they are not applicable in evaluating local aerosol toxicity in the respiratory tract. An often-quoted example is that of diacetyl, a buttery flavor that is commonly used in food products and GRAS for oral consumption (Hallagan, 2017), which is extremely toxic via chronic inhalation exposure causing bronchiolitis obliterans–a lung specific and irreversible respiratory disease (Kreiss et al., 2002; Wallace, 2017). An increasing number of studies suggest that e-cigarettes or e-liquids may cause respiratory tissue damage via inflammation and immunogenic effects and that flavor ingredients may contribute to their toxicity (Higham et al., 2016; Martin et al., 2016; Kaur et al., 2018; Hua et al., 2019). In the United States, all tobacco products marketed after 15 February 2007 must seek market authorization from the FDA. Given the relatively new presence of ENDS, FDA recommends submission of Pre-Market Tobacco Applications (PMTAs) to show whether permitting such new tobacco product to be marketed is appropriate for the protection of the public health, which includes potential health risk to the individual and the population as a whole.

Ideally, e-vapor toxicity assessments should address whether flavor ingredients individually, or as mixtures, cause adverse effects to the respiratory tract upon repeated exposures. The classical approach to evaluate the toxicity of flavor ingredients requires a series of *in vitro* and *in vivo* studies to fill data gaps for individual flavor ingredients and to determine acceptable use levels. However, considering the large number of flavor ingredients available and the even greater number of possible combinations of mixtures in e-liquids (Zhu et al., 2014), the standard approach requires years of testing and thousands of laboratory animals. To add complexity to investigating health effects of e-vapor products, there are no harmonized, preclinical testing methodologies, including e-vapor aerosol generation and characterization, making comparison of findings from different studies difficult (Orr, 2014; Royal College of Physicians, 2016; Breland et al., 2017; McNeill et al., 2018; National Academies of Sciences, 2018; Noël et al., 2018).

In this paper, we propose an alternative approach to evaluate to toxicity of flavors via inhalation, utilizing the “read-across” and a “flavor toolbox” concepts. Instead of testing individual flavor ingredients, we assign each flavor ingredient to a structurally related group (“read-across”), rank and select the “Flavor Group Representatives (FGRs)” based on defined criteria and create mixtures of FGRs (to represent the “flavor toolbox”) for subsequent confirmatory *in vitro* or *in vivo* toxicity testing. This concept was presented at scientific meetings (Sciuscio et al. (2021); Marescotti et al., 2021; Supplementary Resource S1).

## 2 Toxicological Assessment of Flavor ingredients: Structural Grouping Approach

In this “flavor toolbox” approach, available experimental and *in silico*-predicted toxicological information is evaluated to select the FGR as the most biologically active or potentially toxic flavor ingredient within each structural group. The underlying principle is that the biological responses of the most toxic chemical from the group would exceed (or at least be comparable to) the expected toxicity profile of all other chemicals within the same group. Thus, once established, the toxicological data acquired on FGRs could be used to predict the toxicity of structurally-related flavor ingredients and set acceptable use levels (AULs). This concept is not new and follows the principle of the “read-across” approach, where available data from a “data-rich” substance (the source or FGR chemical) is used to evaluate the toxicity of a structurally similar “data-poor” substances (the target or new flavor ingredients).

**FIGURE 2:**
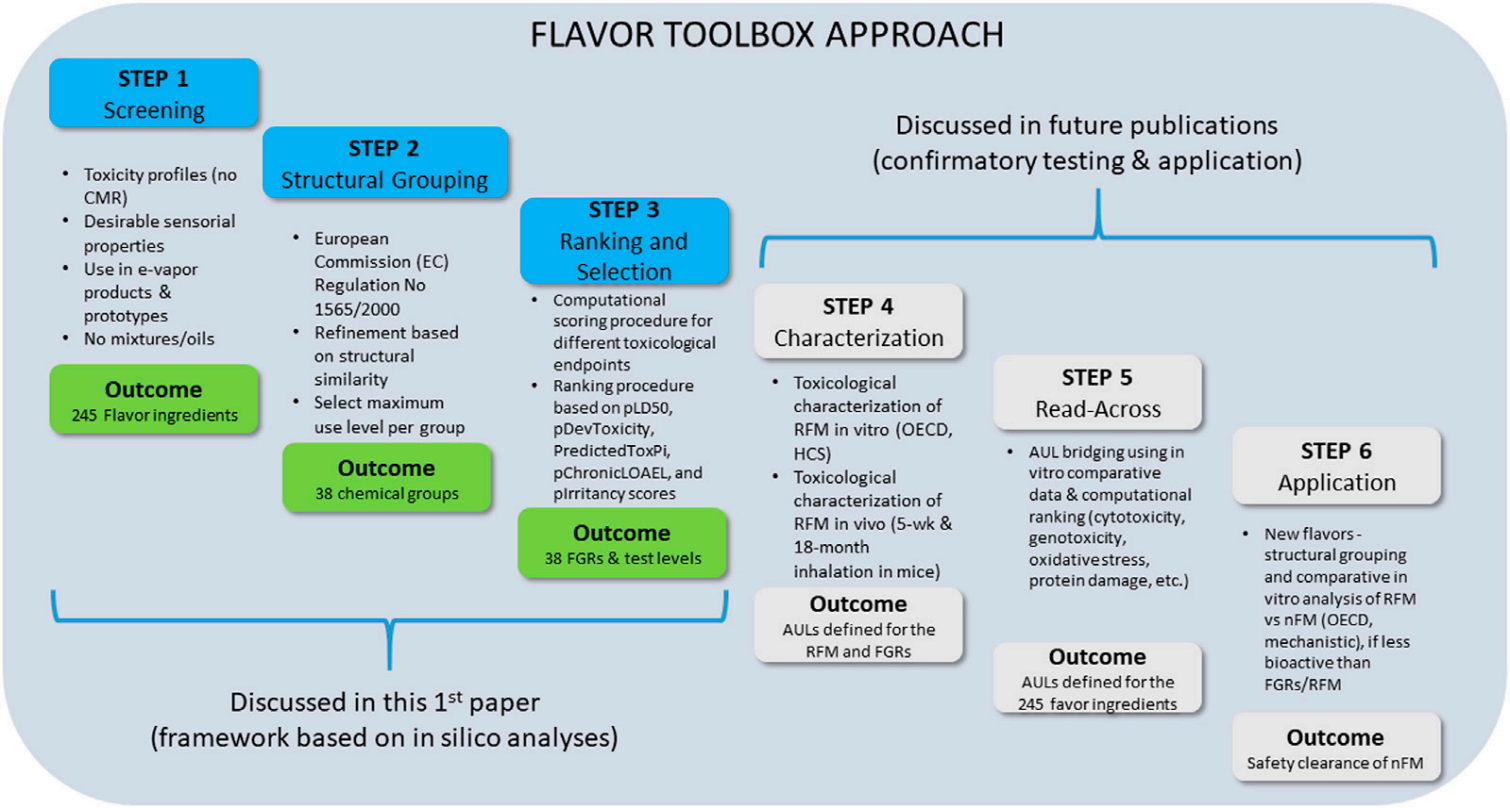
Step by step description of the “flavor toolbox” approach (*Disclosures at scientific meetings, see Supplementary Resource S1)

Figure 2 summarizes all of the steps included in this flavor toolbox approach, including the concept, approach, and a case study of FGR selection, followed by confirmatory toxicity testing of FGR mixtures by *in vitro* and *in vivo* studies. Depending on the test outcomes, the approach may involve additional evaluations before the flavor toolbox can be fully applied to other flavor ingredients and mixtures.

As a case study, this paper follows a flavor mixture through each step of the flavor toolbox approach. The first step is screening all candidate flavor ingredients for known toxicity profiles, desirable sensorial properties, their use in marketed e-vapor products, and historical use in cigarettes. To minimize risks from potential contaminants, the candidate flavor list should include only high-purity USP or food-grade ingredients and exclude complex natural flavor mixtures. In addition, the list should exclude known respiratory sensitizers and carcinogenic, mutagenic, or reprotoxic (CMR) compounds (e.g., ingredients classified as group 1, 2A, or 2B carcinogens by the International Agency for Research on Cancer (IARC) or classified as CMR by other authoritative bodies). Once selected, the toxicology data (*in vitro* and *in vivo*) available from the literature is evaluated for each flavor ingredient. In the case of data gaps, computational toxicology software could be used to obtain predicted toxicological properties, and additional *in vitro* high-content screening (HCS) data to characterize the mode of action of flavor ingredients can be collected (Marescotti et al., 2016; Marescotti et al., 2020).

The second step of the flavor toolbox approach is to assign or group each flavor ingredient based on their structural, toxicological, and metabolic properties. A well-known example of this approach has been used by the European Food and Safety Authority (EFSA) to evaluate flavor ingredients for their acceptability for use in foods. The EFSA’s Flavorings Group Evaluation (FGE) approach has defined groups of flavor ingredients with similar structural and metabolic properties based on the grouping strategy detailed in Commission Regulation No. 1565/2000 (European Commission, 2000). Similarly, Date et al. (2020) developed a tiered system for chemical classification of fragrances on the basis of (a) organic functional group, (b) structural similarity and reactivity features of the hydrocarbon skeletons, (c) predicted or experimentally veriﬁed phase I and phase II metabolism, and (d) expert screening to consider these variables in the context of speciﬁc toxicity endpoints (Date et al., 2020).

The third step is to select the flavor that can represent each group (i.e., FGRs). This can be achieved by using a scoring system to rank each flavor chemical within a group on the basis of the available experimental and predicted toxicological data. This requires objective and quantitative scoring metrics and a computational procedure to calculate the composite toxicity score in order to select the representative flavor—the flavor ingredient with the highest predicted toxicity score—for each flavor group. This paper explains details of the first three steps in the flavor toolbox development using a case study flavor mixture. For completeness, below we briefly explain the remaining steps, including feasibility and confirmatory *in vitro* and *in vivo* testing, as outlined in Figure 2.

The fourth step is to create a representative flavor mixture (RFM) including all FGRs representing the flavor toolbox. This RFM is then subjected to confirmatory *in vitro* or *in vivo* studies. In particular, *in vitro* toxicity and mechanistic investigations provide a better understanding of hazards associated with the RFM and may potentially yield *in vitro* Point of Departure (PoD) estimates. From *in vivo* chronic inhalation toxicity studies, a benchmark dose (BMD10) or ‘no observed adverse effect level’ (NOAEL) of the RFM could be obtained and ultimately used for inhalation risk assessment to support the AULs for all FGRs in the flavor toolbox.

The fifth step is to demonstrate the “read-across” approach for all other flavor ingredients (245) included in the toolbox list. This is achieved by performing a series of *in vitro* HCS studies on cytotoxicity, genotoxicity, and other toxicological endpoints (e.g., oxidative stress, protein damage etc.) on each flavor ingredient and comparing the results, qualitatively (mechanism) and quantitatively (potency or PoD) with its FGR. If a given flavor ingredient causes similar toxicological perturbations that are less than/equal to those of the FGR in the same flavor group, the AUL set for the FGR could be applicable as well. In contrast, if a flavor ingredient of a specific group causes different or greater toxicological perturbations than its corresponding FGR, the FGR AUL cannot be directly applied; additional *in vitro* and *in vivo* studies may be necessary and depending on the biological response, the flavor may potentially be excluded from the flavor toolbox.

The sixth and final step is to independently confirm the flavor toolbox approach, by creating and testing “new” flavor mixtures (nFMs) of any flavor ingredients in the full list, such as an nFM candidate that is evaluated for e-vapor product development. This step is important to account for potential interactions that may occur between different flavor ingredients selected for inclusion in the nFM. Therefore, any candidate flavor mixture should be screened for *in vitro* cytotoxicity, genotoxicity and other defined toxicological endpoints from the above testing framework and compared with the RFM tested *in vitro*. In the case of nFMs that induce greater or different biological responses compared to the RFMs, the outcomes from the above flavor toolbox chronic *in vivo* studies and their NOAELs would not be directly applicable.

## 3 A Case Study: Applying the Flavor Toolbox Approach

In this section we provide an example of how the flavor toolbox approach described above can be applied.

### 3.1 Generation of a List of Flavor Ingredients for E-Vapor Products and Toxicological Evaluation

The first step of the flavor toolbox approach is to select a list of candidate flavor ingredients and evaluate their toxicity profiles for potential ranking. In this case study, 245 commonly used individual flavor ingredients were selected and their available toxicological data were collected using both the (European Chemicals Agency, 2002)) and (Toxplanet, 2021) databases. If there were multiple values for the same toxicological endpoint (e.g., LD50), the ECHA approach was applied and the most relevant data set was selected as follows: in the context of REACH (Registration Evaluation Authorization and Restriction of Chemicals), evaluation of data quality includes an assessment of the adequacy of the information for hazard/risk assessment as well as for classification and labelling (C&L) purposes and elements of relevance and reliability. The ECHA data categorization for the selection of data from reliable “key studies” only (Klimisch et al., 1997) was used whenever possible. A study was considered a reliable “key study” if it was generally expected to be the most adequate, reliable, and relevant for a specific element/endpoint study section. If properly reported, a key study may fulfil a REACH information requirement on its own. At the same time, we confirmed that all 245 flavor ingredients were not classified as respiratory sensitizers or as group 1, 2A, or 2B carcinogens in the IARC classification or CMR by the FDA.

**FIGURE 3:**
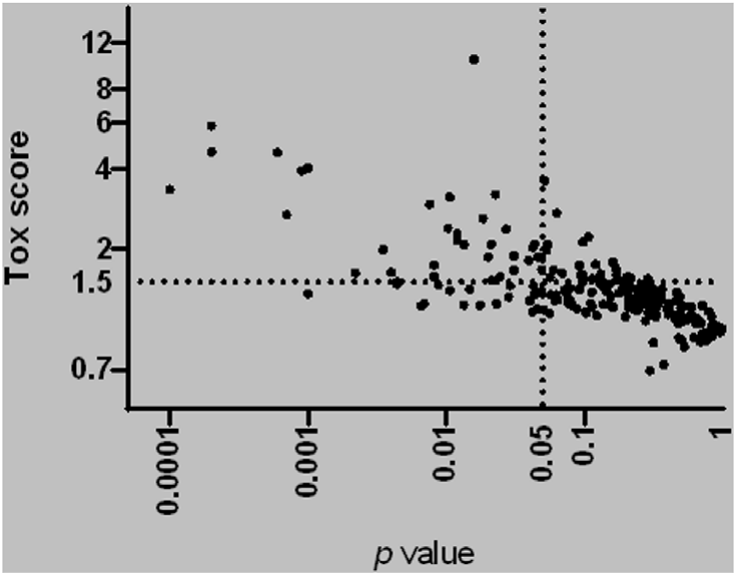
Tox-score for all individual flavor ingredients tested using real-time cell analysis. The horizontal straight line indicates a 1.5 EC50 ratio (ratio of EC50 base solution/EC50 flavored solution). The vertical straight lines indicate a p-value equal to 0.05 on the right. Each dot corresponds to one individual flavor ingredient.

During the review of available data, we found that most individual flavor ingredients lacked experimental toxicity data. Therefore, additional *in vitro* testing was performed on all 245 individual flavor ingredients for cytotoxicity screening by real-time cellular analysis (RTCA). Figure 3 summarizes the Tox Score (ratio of the EC50 of the base solution to the EC50 of the flavored solution) versus the *p* values for all individual flavor ingredients tested. As described previously (Marescotti et al., 2020), we applied a statistically significant Tox Score value of 1.5 to identify the most cytotoxic flavor ingredients in the list and selected a total of 34 flavor ingredients for subsequent *in vitro* characterization of their mode of action using HCS analysis.

**FIGURE 4:**
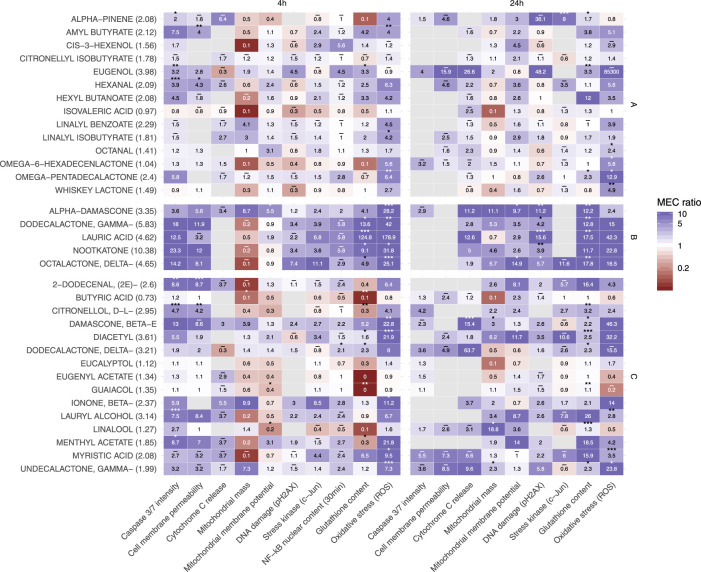
Heatmaps of the HCS endpoints impacted by single flavor ingredients tested after 30 min (only for NF-kB endpoint), 4 and 24 H exposure.

In the HCS assessment (Table 1), Normal Human Bronchial Epithelial (NHBE) cells were exposed in a concentration-dependent manner to 34 individual flavor ingredients and to non-flavored (base) matrix for either 4 h or 24 h (30 min for the NF-kB translocation endpoint.) (Gonzalez-Suarez et al., 2016; Marescotti et al., 2016) Results are shown in Figure 4 in three heatmaps which grouped these flavor ingredients based on minimal effective concentration (MEC) profile similarities and the Tox or Phenotypic Score (ratio of MEC base solution/MEC flavored solution) is shown in Table 2.

**TABLE 1:** HCS endpoints description.

Assays	#	Biological Endpoint	Description
All assays	1	Cell Count	A decreasing number of cells indicates toxicity due to necrosis, apoptosis, or a reduction in cellular proliferation
Cytotoxicity Screening Panel	2	Mitochondrial Mass	A decrease in mitochondrial mass indicates a loss of total mitochondria. An increase in mass implies either mitochondrial swelling or an adaptive response to cellular energy demands
	3	Mitochondrial Membrane Potential	A decrease indicates mitochondrial toxicity as well as a potential role in apoptosis signaling. An increase indicates an adaptive response to cellular energy demands
	4	Cytochrome C Release	An increase is one of the hallmarks of the apoptosis signaling cascade
DNA Damage	5	Phospho-Histone 2AX	Histone H2AX phosphorylation occurs following the induction of DNA double-strand breaks [it correlates with neutral comet assay results (Li et al., 2006)]
Oxidative Stress	6	Reactive Oxygen Species (ROS)	Assay uses the redox indicator dihydroethidium (DHE). An increase in signal indicates increase ROS formation
Glutathione Content	7	Glutathione (GSH)	The dye used (monochlorobimane) forms a fluorescent GSH adduct catalyzed by GSH-S-transferase. A decrease in signal indicates a decrease in cellular levels of GSH and suggests the presence of oxidative stress
Apoptosis/Necrosis	8	Caspase 3/7 Activity	An increase indicates the onset of the cell signaling apoptosis/necrosis cascade leading to cell death by apoptosis
	9	Cell Membrane Permeability	An increase is a general indicator of cell death
Stress Kinase	10	Phospho c-jun	An increase indicates the upregulation of the stress kinase pathway, which includes downstream targets such as cell differentiation and apoptosis
NF-kB	11	NF-kB Nuclear Translocation	An increase in signal indicates that the transcription factor NF-kB is recruited to the cell nucleus to activate the expression of its target genes. Downstream effects include inflammation, cell survival, or apoptosis pathways.

**TABLE 2:** Phenotypic scores (ratio of MEC base solution/MEC flavored solution) and HCS endpoints counts (number of endpoints which returned a computed MEC; higher is the number the more biological pathways are activated) for the 34 individual flavor ingredients that were evaluated using an HCS approach. Diacetyl is not included in the list of flavor ingredients and was added here only as a positive control.

	Flavor ingredients	Tested conc. (ppm)	Phenotypic score	HCS endpoints (count)
A	Eugenol	10,800	5,74	16
	Citronellyl Isobutyrate	1,140	4,41	6
	Linalyl Benzoate	540	4,34	9
	Omega Pentadecalactone	415	3,59	12
	Alpha Pinene	225	3,14	12
	Amyl Butyrate	2,700	2,92	14
	Hexen-1-OL-3	30,000	2,43	12
	Hexyl Butanoate	2,160	2,40	12
	Linalyl Isobutyrate	1,080	2,11	15
	Hexanal	2,160	1,77	17
	Octanal	540	1,00	19
	Whiskey Lactone	3,735	0,91	15
	Valeric Acid	42,000	0,77	15
	Omega 6-Hexadecenlactone	400	0,66	21
B	Lauric Acid	4,500	13,36	14
	Delta Octalactone	31,125	9,43	15
	Gamma Dodecalactone	3,192	8,94	14
	Nooktanone	4,500	7,39	14
	Alpha Damascone	1,080	5,88	18
C	Gamma Undecalactone	1,992	5,24	17
	Beta E-Damascone	679	5,00	18
	*Diacetyl 1% (Positive Control)	10	4,35	16
	Menthyl Acetate	2,300	3,27	13
	(2E)-2-Dodecenal	104	3,25	13
	Beta Ionone	1,440	3,21	18
	Citronellol	4,800	3,16	12
	Myristic Acid	450	2,88	13
	Lauryl Alcohol	270	2,78	15
	Delta Dodecalactone	2,988	2,12	18
	Linalool	2,400	1,29	13
	Eucalyptol	720	1,08	14
	Guaiacol	107	0,84	15
	Eugenyl acetate	1440	0,78	16
	Butyric acid	5600	0,71	16

Flavor ingredients in heatmap “A” had the lowest impact for most of the HCS endpoints tested, which also fits with their lower Tox Score as well as with their lower phenotypic score (Table 2). Flavor ingredients in heatmap “B” showed the highest and most significant impact for reactive oxygen species (ROS) and glutathione (GSH) content endpoints after 4 h exposure and for DNA damage and GSH content endpoints after 24 h exposure (Figure 4). Also, for this group, no ratio was computed for the cytochrome c release endpoint at 4 h (except alpha damascone) or the cell membrane permeability endpoint at 24 h. For heatmap “B,” the range of phenotypic scores was between 5.88 and 13.36 (Table 2). Flavor ingredients in heatmap “C” had a phenotypic score ranging between 0.71 and 5.24 (Table 2).

In addition to the experimental data, TOPKAT^®^ (Toxicity Prediction by Komputer Assisted Technology, 2017) prediction software was used to acquire predicted toxicological information for all 245 flavor ingredients. TOPKAT contains models covering a range of different toxicity endpoints including rat chronic LOAEL, irritancy, developmental toxicity, rodent carcinogenicity, carcinogenicity potency TD50, and other endpoints. Many of the models provide a binary summary prediction (yes or no), whereas other models, such as those for acute oral toxicity, estimate a LD50 value. Additionally, Cramer classes were assigned to each flavor ingredient using profilers available in the QSAR toolbox software (OECD QSAR, 2019). Cramer classes have been proposed to support the Threshold of Toxicological Concern (TTC) for inhalation toxicity and chemicals assigned to Cramer class I are deemed less toxic than those assigned to Cramer classes II or III both at local (respiratory) and systemic levels (Carthew et al., 2009; Costigan and Meredith, 2015). For example, the application of the Cramer classification tree to a data set of 92 subacute or subchronic rat inhalation studies resulted in TTC values of 1400 (class 1) and 470 µg/person/day (class III) for local effects (based on human lung weight of 650 g) and of 980 (class 1) and 170 µg/person/day (class III) for systemic effects (Carthew et al., 2009). Although these TTC values have been proposed to aid in toxicological risk assessment of flavor ingredients in e-liquids (Costigan and Meredith, 2015), in this case study we did not use TTCs as the limit for AULs. Instead, we used the TTC as well as *in silico* data to rank flavor ingredients for their toxicity potential for the subsequent FGR selection. Supplementary Resources S2, S3 show the summary of the obtained literature/experimental (Group 1–2B) toxicity data sources and the *in silico* predicted toxicological tools and endpoints.

### 3.2 Grouping Flavor Ingredients

The second step of the flavor toolbox approach is to assign each individual flavor ingredient into the appropriate flavor group based on their structural, toxicological, and metabolic properties. The 245 flavor ingredients selected in this case study were initially allocated to one of the 34 structural groups defined in the European Commission (EC) Regulation No 1565/2000 (European Commission, 2000). Some groups (e.g., EC groups 1 and 2) contain many flavor ingredients (primary aliphatic alcohols/aldehydes/acids, acetals, and esters) and these broader, heterogeneous, structural groups were further subdivided to better represent the range of structural differences. Following this approach, a total of 38 groups were defined, encompassing 27 of the original 34 EC groups (Table 3).

**TABLE 3:** Chemical groups for flavor ingredients defined in the commission regulation (EC) No 1565/2000 and further subclustering and selected FGRs for this case study.

(EC) No 1565/2000 Group	Description	Sub-grouping in this case study	Flavor group representatives (FGRs)
Group 1	Straight-chain primary aliphatic alcohols/aldehydes/acids, acetals and esters with esters containing saturated alcohols and acetals containing saturated aldehydes, No aromatic or heteroaromatic moiety as a component of an ester or acetal	Group 1	Acetal
		Group 1–2 a	Isobutyraldehyde
Group 2	Branched-chain primary aliphatic alcohols/aldehydes/acids, acetal and esters with esters containing branched-chain alcohols and acetals containing branched-chain aldehydes, No aromatic or heteroaromatic moiety as a component of an ester or acetal	Group 1–2 b	Isoamyl alcohol
		Group 1–2 c	2-methylbutyric acid
		Group 1–2 d	Ethyl 2-methylbutyrate
Group 3	α, β-unsaturated (alkene or alkyne) straight-chain and branched-chain aliphatic primary alcohols/aldehydes/acids, acetals and esters with esters containing α, β-unsaturated alcohol and acetal containing α, β-unsaturated alcohols or aldehydes, No aromatic or heteroaromatic moiety as a component of an ester or acetal	Group 3	(*E,z*)-2,6-nonadienal
		Group 3–4	D-l-citronellol
Group 4	Non-conjugated and accumulated unsaturated straight-chain and branched-chain aliphatic primary alcohols/aldehydes/acids, acetals and esters with esters containing unsaturated alcohols and acetals containing unsaturaed alcohols or aldehydes, No aromatic or heteroaromatic moiety as a component of an ester or acetal	Group 4	Cis-3-hexenol
Group 5	Saturated and unsaturated aliphatic secondary alcohols/ketones/ketals/esters with esters containing secondary alcohols, No aromatic or heteroaromatic moiety as a component of an ester or ketal	Group 5 a	Isopulegol
		Group 5b	1-penten-3-one
Group 6	Aliphatic, alicyclic and aromatic saturated and unsaturated tertiary alcohols and esters with esters containing tertiary alcohols, Esters may contain any acid component	Group 6	Linalool
Group 7	Primary alicyclic saturated and unsaturated alcohols/aldehydes/acids/acetals/esters with esters containing alicyclic alcohols, Esters/acetals may contain aliphatic acyclic or alicylic acids or alcohol component	N/A	N/A
Group 8	Secondary alicyclic saturated and unsaturated alcohols/ketones/ketals/esters with ketals containing alicyclic alcohols or ketones and esters containing secondary alicyclic alcohols, Esters may contain aliphatic acyclic or alicyclic acid component	Group 8 a	Alpha-damascone
		Group 8 b	Piperitone
Group 9	Primary aliphatic saturated or unsaturated alcohols/aldehydes/acids/acetals/esters with a second primary, secondary or tertiary oxygenated functional group including aliphatic lactones	Group 9 a	Delta nonalactone
		Group 9 b	Ethyl lactate
		Group 9 c	Triethyl citrate
Group 10	Secondary aliphatic saturated or unsaturated alcohols/ketones/ketals/esters with a second secondary or tertiary oxygenated functional group	Group 10	3-methyl-2,4-nonanedione
Group 11	Alicyclic and aromatic lactones	Group 11	Dihydroactinidiolide
Group 12	Maltol derivatives and ketodioxane derivatives	Group 12	Ethyl maltol
Group 13	Furanones and tetrahydrofurfuryl derivatives	Group 13	Furaneol
Group 14	Furfuryl and furan derivatives with and without additional side-chain substituents and heteroatoms	N/A	N/A
Group 15	Phenyl ethyl alcohols, phenylacetic acids, related esters, phenoxyacetic acids and related esters	Group 15	2-methyl-4-phenyl-2-butanol
Group 16	Aliphatic and alicyclic ethers	Group 16	Ambrox
Group 17	Propenylhydroxybenzenes	N/A	N/A
Group 18	Allylhydroxybenzenes	Group 18	Eugenyl acetate
Group 19	Capsaicin related substances and related amides	N/A	N/A
Group 20	Aliphatic and aromatic mono- and di- thiols and mono-, di-, tri-, and polysulfides with or without additional oxygenated functional groups	Group 20	P-mentha-8-thiol-3-one
Group 21	Aromatic ketones, secondary alcohols and related esters	Group 21	Acetanisole
Group 22	Aryl-substituted primary alcohol/aldehyde/acid/ester/acetal derivatives, including unsaturated ones	Group 22	Methyl cinnamate
Group 23	Benzyl alcohols/aldehydes/acids/esters/acetals, Benzyl and benzoate esters included, May also contain aliphatic acyclic or alicyclic ester or acetal component	Group 23 a	Ethyl vanillin
		Group 23 b	Benzyl alcohol
Group 24	Pyrazine derivatives	Group 24	2,5-dimethylpyrazine
Group 25	Phenol derivatives containing ring-alkyl, ring-alkoxy, and side-chains with an oxygenated functional group	Group 25	2-methoxy-4-methylphenol
Group 26	Aromatic ethers including anisole derivatives	Group 26	Para-dimethoxybenzene
Group 27	Anthranilate derivatives	Group 27	Methyl anthranilate
Group 28	Pyridine, pyrrole, and quinoline derivatives	Group 28 a	3-ethylpyridine
		Group 28 b	2-acetylpyrrole
Group 29	Thiazoles, thiophene, thiazoline and thienyl derivatives	Group 29	2-acetylthiazole
Group 30	Miscellaneous substances	Group 30	Ketoisophorone
Group 31	Aliphatic and aromatic hydrocarbons	Group 31 a	Alpha-pinene
		Group 31 b	Para-cymene
Group 32	Epoxides	N/A	N/A
Group 33	Aliphatic and aromatic amines	N/A	N/A
Group 34	Amino acids	N/A	N/A

### 3.3 Selection Flavor Group Representatives

The third step of the flavor toolbox approach is to select the flavor ingredients that best represent each flavor group. This step can be achieved by ranking each of the flavor ingredients within each flavor group on the basis of the available experimental and predicted toxicological data and subsequently applying a numerical scoring and a computational procedure to select the FGR, the flavor ingredient with the predicted highest toxicity potential within its flavor group.

**FIGURE 5:**
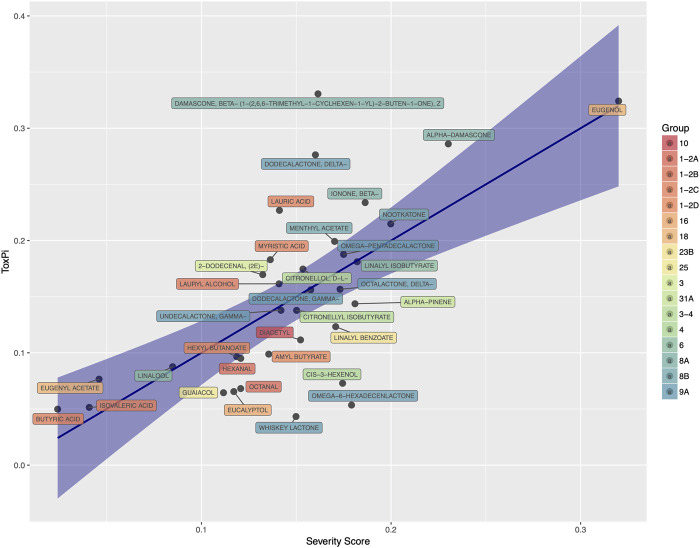
Predicted ToxPi index (Called Severity Score) as predicted by the slected regression model *vs*. experimental ToxPi index. The R value of this model is 0.67. FGRs are colored by chemical groups.

A numerical score (code) for toxicological attributes (if available) was assigned as follows:

• pCramer: Cramer class coded 0, 1 or 2 (for Cramer class I, II, and III, respectively).
• pIrritancy, pCarcinogenicity and pDevToxicity: logical TOPKAT predictions, scored 1 (true) or 0 (false).
• pExpCarcinogenicity: carcinogenicity defined as the sum of mutagenicity and genotoxicity, which were individually scored “Negative”, “Equivocal”, or “Positive” based on available experimental data. Numerically scored as 0, 1/2, and 1, respectively.
• pXCelligence: experimental ratio of the EC50 for the base matrix and the flavor ingredient EC50 (Tox Score) based on real-time cell analysis and HCS *in vitro* assays. This continuous score was transformed into ranks across all flavor ingredients.
• pLC50: predicted acute inhalation toxicity using TOPKAT. This continuous score was transformed into ranks across all flavor ingredients.
• pChronic_LOAEL: predicted chronic LOAEL from TOPKAT. This continuous score was transformed into ranks across all flavor ingredients.
• pNOAEL, pExpLC50, pLD50: multiple evidence (NOAEL, LC50, LD50) extracted from literature and public database:
  • Two available features were summarized as follows (e.g. f1 = LD50 from ECHA LD50 and f2 = Toxplanet LD50 in rodents): f1 by default; if f1 missing use f2; if f1 or f2 “>…” take the worst-case scenario (lowest LD50).
  • The normalized rank of the extracted “-feature” across all flavor ingredients was subsequently used
• pToxPi: predicted ToxPi index. The ToxPi index is a numerical index developed by the EPA (Reif et al., 2013) that can be used for ranking, using multiple domains of information (in our case HCS assay endpoints). The ToxPi index is defined as a weighted sum of the phenotypic score; phenotypic scores were obtained from HCS experiments with 34 flavor ingredients at the time of this analysis (see Section 3.1). In order to complement this attribute for all flavor ingredients, a prediction model was developed. After statistical feature selection, pCramer, pIrritancy, pChronicLOAEL, pExpCarcinogenicity and pXCelligence were retained in the final model; while pLC50, pCarcinogenicity, and pDevToxicity were excluded as predictors. Figure 5 shows the accuracy of the prediction model in that for a subset of flavor ingredients, both the predicted and experimental ToxPi indices were correlated with the correlation R of 0.67. Applying the final model, the ToxPi indices for all flavor ingredients were then predicted and ranked.

In order to select FGRs, flavor ingredients within each group were ranked based on pLD50, pDevToxicity, PredictedToxPi, pChronicLOAEL and pIrritancy scores. For each flavor ingredient, the average rank was then computed and used to generate the final ranking. The resulting FGRs are listed in Table 3 and Supplementary Resource S3 details the ranking process using one example (group 1–2B).

### 3.4 Representative Flavor Mixture Characterization

The fourth step of the flavor toolbox approach is to create a mixture as a proxy to the full “flavor toolbox” by combining all FGRs and assess its toxicity. This RFM should be tested *in vitro* and *in vivo* in order to characterize both the hazards associated with the RFM and to define the *in vivo* benchmark dose or NOAEL. Below is the recommended plan for these preclinical studies. Some of preliminary findings for the case study mixtures were presented at scientific meetings (Supplementary Resource S1), as briefly summarized.

#### 3.4.1 Assembling of RFM and Flavor Concentrations

In assembling the RFM for *in vitro* and *in vivo* studies, several factors should be considered when determining the testing concentration of FGRs. In particular, the concentrations of FGRs in RFMs should cover the projected consumer exposure during typical e-vapor usage, taking into account, for instance, information from marketed products and/or the sensorial properties of each. Moreover, in order to evaluate the wide range of possible toxic effects *in vivo* studies, groups of animals should be exposed to both the human relevant (low) concentrations of FGRs, as well as the exaggerated or maximum tolerated (high) concentrations, based on solubility and technical feasibility limits. In addition, the levels of other ingredients typically included in e-liquid (carriers and nicotine) should be defined and the resulting final formulations tested for stability for the duration of usage.

#### 3.4.2 Characterization and Stability of RFM and Aerosols

**FIGURE 6:**
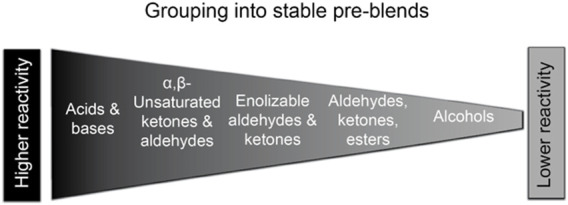
Criteria for generating concentrated pre-blend formulations.

Briefly, in order to maximize stability and simplify the preparation of RFM for long-term inhalation studies, we created a limited number of stable, concentrated flavor mixtures (these “pre-blends” did not contain nicotine and carriers). A total of five pre-blends were prepared on the basis of chemical structure, solubility, and chemical reactivity of flavor ingredients (i.e., unreactive, electrophilic, nucleophilic, basic, and acidic) (Figure 6). The stability of these pre-blends were first confirmed by individual flavor analysis for up to 2 weeks (Smith et al., 2019). All pre-blends were then mixed with the remaining ingredients (e.g., nicotine and carriers) to make the final e-liquid formulations. The stability of the final RFMs were shorter (<1 week) for the case study flavor mixtures (Smith et al., 2019) and, therefore, the RFMs should be prepared fresh, using pre-blends at least twice per week for long-term inhalation exposures.

In addition to stability, the aerosolization of e-liquid formulations and the e-liquid-to-aerosol transfer rates of ingredients should be characterized prior to *in vivo* inhalation testing. For the case study RFMs discussed in this paper, we presented the preliminary results of aerosol characterization at a scientific conference in 2019 (Zhang et al., 2019). Briefly, we used a device-independent aerosol generator, a CAG (Capillary Aerosol Generator) with the capillary temperature set at 250°C, which is within the range of typical temperatures of e-cigarette coils (Geiss et al., 2016). In the case study RFMs, flavor analysis of the generated aerosol was determined by collecting aerosol with a filter pad followed by a series of impingers containing ethanol. The major ingredients of the RFMs (PG, VG, nicotine) were analyzed in addition to aerosol pH and the particle size distribution. Results confirmed that the ingredient content and the pH of aerosol were consistent with the e-liquid, and the mass median aerodynamic diameter (MMAD) of the aerosol was around 1 µm with the geometric standard deviation (GSD) < 2 (Zhang et al., 2019). Additionally, the yield of six representative flavor ingredients from the RFM e-liquid-to-aerosol was quantified in an *in vivo* exposure chamber (Wong et al. in prep). For the case study RFMs discussed in this paper, we presented the preliminary results of the stability assessment at a scientific conference in 2019 (Smith et al., 2019).

#### 3.4.3 In *vitro* Characterization of FGRs e-formulations

In addition to the individual ingredient *in vitro* assays discussed earlier, the final RFMs should be evaluated for biological activity as mixtures using standard and/or mechanistic *in vitro* assays. While the ultimate aerosol toxicity assessment will be based on *in vivo* inhalation studies, the *in vitro* results will help understand the potential toxicity of the final RFMs in terms of specific (e.g., genotoxicity) hazard as well as underlying mechanisms. The standard *in vitro* assays include the Ames bacterial mutagenicity test, the Mouse Lymphoma Assay (MLA), an *in vitro* micronucleus genotoxicity assay (MNvit), and the neutral red uptake (NRU) assay for cytotoxicity (INVITTOX, 1990; Organization for Economic Cooperation and Development, 1997; Organization for Economic Cooperation and Development, 2016a; Organization for Economic Cooperation and Development, 2016b). In addition to the standard *in vitro* assays, a mechanistic *in vitro* assay that characterizes the genotoxic mode of action of RFMs (e.g., ToxTracker^®1^) can be considered. The ToxTracker^®^ assays consist of six reporter cell lines, which are developed for unique biomarkers that may discriminate between induction of DNA damage, oxidative stress, protein damage, and general cellular stress. The *in vitro* results obtained in this step can serve as a basis for the subsequent head-to-head comparison in the final step of the flavor toolbox approach as described in Section 4.4.6.

#### 3.4.4 I*n vivo* Characterization of FGRs Aerosols

In 2018 the US National Academies of Sciences pointed out the lack of chronic inhalation safety data and recommended conducting “long-term animal studies, using inhalation exposure to e-cigarette aerosol, to better understand risks from inhaling complex mixtures containing reactive carbonyl compounds, flavor ingredients and additives” (National Academies of Sciences, 2018). Among reported animal models, we recommend the A/J mouse model for the investigation of chronic toxicity and carcinogenic potential of the RFMs. In contrast to other rodent models, the A/J mouse is highly susceptible to lung tumor induction and has been used as a screening model in carcinogenicity testing and chemoprevention studies (Monteillier et al., 2018). In addition, past A/J mouse studies have successfully demonstrated the development of lung tumors following up to 18-months mainstream cigarette smoke (CS) exposure (Stinn et al., 2010; Stinn et al., 2013a; Stinn et al., 2013b). In addition to CS-induced lung tumors, the A/J models showed pronounced lung inflammation accompanied by emphysematous changes, indicating that the A/J mouse is also a suitable model for smoking-induced emphysema and chronic obstructive pulmonary disease (COPD) (Stinn et al., 2013a; Stinn et al., 2013b; Cabanski et al., 2015; Wong et al., 2020). The study endpoints should include not only phenotypic microscopic evaluation (lung tumor incidences and severity), but also systems toxicology assessment to aid in potentially differentiating between test article-induced lung tumors and spontaneously arising tumors (Luettich et al., 2014; Titz et al., 2020).

### 3.5 Estimating Acceptable use Levels for Toolbox Flavor Ingredients not Included in the Tested RFMs

The fifth step of the flavor toolbox approach is to confirm AULs for the remaining flavor ingredients included in the initial list. This step is applicable after the completion of the chronic *in vivo* studies of the RFMs and the confirmation of NOAELs based on evaluation of chronic toxicity outcomes. We hypothesize the chronic *in vivo* inhalation data would allow the determination of the benchmark dose or NOAEL (or LOAEL) based on systemic and respiratory toxicity and carcinogenicity outcomes of RFMs. Then, AULs for the rest of flavor ingredients within a given chemical group could be matched to the AUL of its respective FGR based on the ‘read-cross’ principle that flavor ingredients within a group have an equal/lower biological activity than the respective FGR. As discussed earlier, this read-cross step should be accompanied by independent mechanism-based HCS *in vitro* studies for all flavor ingredients against the selected FGRs. The outcomes of these *in vitro* studies should confirm that the biological activity across all flavor ingredients within a chemical group are qualitatively comparable and that the selected FGRs are indeed the most biologically active (toxic) ingredient in the respective chemical groups. Following these *in vitro* and *in vivo* confirmatory studies, the NOAELs of RFMs could be applied to the remaining flavors in the toolbox list.

### 3.6 Application of Flavor Toolbox Approach to New Formulations

After the above steps are executed according to the proposed rationale and assumptions, the sixth and the last step of the flavor toolbox approach is to explore the approach applied to new flavor mixtures. In this step, a hypothetical new flavor mixture (nFM) can be generated using any flavors in the toolbox list and the AULs defined in the above sections. This nFM should be tested for the battery of *in vitro* OECD and mechanistic assays suggested above, and the results compared to the corresponding *in vitro* testing of the specific RFM tested in chronic *in vivo* studies at the NOAEL or LOAEL level. The *in vitro* testing would allow an indirect screening of unexpected, synergistic, or adverse responses from the nFM mixtures. If the comparison of *in vitro* battery results shows that the nFM has toxicity lower than/equal to that of the RFM, then the nFM could be considered for subsequent product development. Over time, the cumulative results of this approach would help expand the toolbox database, inform potential interactions among flavor ingredients, enhance predictability of the flavor “read-across” approach, and limit the need for *in vivo* studies to investigate *in vitro* results.

## 4 Advantages and Limitations of the Flavor Toolbox Approach

In this work, we have presented a pragmatic *in silico* and experimental, data-based flavor toolbox approach that supports the establishment of AULs for flavor ingredients (up to 245 in this case study) and ultimately other new flavor mixtures as part of product development. The approach can be expanded, in theory, to a new individual flavor ingredient (nFI) not initially included in the tested “flavor toolbox” list. This can be done following the fifth step by allocating the nFI into the most appropriate chemical group based on its structure and executing the *in vitro* battery of assays against the FGRs to evaluate the application of the chemical group’s AUL.

While the presented flavor toolbox approach has many strengths, it also has limitations. First, the grouping strategy adopted in this case study (EC Regulation No 1565/2000) is based on a grouping procedure that is developed for flavor ingredients for ingestion, not for inhalation exposures. Therefore, additional *in vivo* inhalation studies were incorporated to validate the approach for the case study flavor mixtures. As additional inhalation data on flavors becomes available, the grouping approach should be reviewed in the context of new data and, if necessary, revised, focusing on biological and metabolic activity in the respiratory tract. Second, the “additivity” assumption of flavor mixtures is a limitation in that each selected FGR is unlikely to contribute equally to the overall toxicity of the RFM. It is possible that the *in vivo* toxicity outcomes and the resulting NOAEL (or LOAEL, BMDL10) of the RFM are primarily driven by the toxicity of most toxic FGRs, and this might lead to overly conservative AULs for other, individually less-toxic FGRs. Finally, the selection of the *in vitro* testing battery is critical to derive AULs for flavor ingredients not tested *in vivo* and to screen nFMs against the RFM. While the *in vitro* HCS battery suggested here measures a wide range of biological activities, it is not meant to preclude the use of other assessment tools. Careful consideration should be given to select an *in vitro* battery that not only includes standard endpoints (e.g., cytotoxicity, mutagenicity, genotoxicity), but other mechanistic and phenotypic endpoints using human cellular systems (e.g., oxidative stress, inflammation, DNA damage, histopathology).

## 5 Discussion

Many flavor ingredients used in RRPs are GRAS for use in food. However, the Flavor and Extract Manufacturers Association has strongly cautioned that their GRAS certification is intended for exposure by ingestion, not inhalation (Hallagan, 2014). Furthermore, with respect to the use of flavored e-vapor product aerosol as RRPs, some studies have reported potential adverse effects of e-vapor product aerosol. For instance, Ghosh et al. (2019) reported elevated neutrophil elastase matrix metalloproteinases activities in bronchoalveolar lavage fluid of both vapers and smokers relative to non-smokers and suggested that e-vapor product aerosol might adversely affect the lung parenchyma. In contrast, other repeated inhalation studies of e-vapor product aerosol exposures have demonstrated reduced risk potential in animal models compared to cigarette smoke (Wong et al., 2020; Kumar et al., 2021). These apparently conflicting results warrant a robust toxicity assessment of flavor ingredients in e-vapor product aerosol via long-term inhalation exposures.

In this work, we presented a pragmatic, structure group-based approach that uses *in silico* predictive toxicity modeling, followed by targeted *in vitro* toxicity and *in vivo* long-term inhalation studies, to evaluate many different flavor toolbox ingredients. Using this approach, we presented a case study where a total of 245 flavor ingredients were assigned to groups of structurally related compounds and 38 FGRs with the predicted worst toxicological profile within each group were identified and combined to generate a RFM, a proxy of the toolbox chemical variety across all 245 ingredients. We have proposed *in vitro* HCS screening and *in vivo* inhalation studies on FGRs and RFMs, with intent to use the resulting data to predict the toxicity of structurally related flavor ingredients and ultimately to assess the toxicity of nFMs.

This concept follows the principle of the “read-across” approach where available data for a “data-rich” substance (the source) are used for the toxicity evaluation of a “data-poor” substance (the target), which is considered similar enough to the source substance. At the same time, two major aspects of the “read-across” exercise should be considered: similarity and uncertainty. While there is rationale for structure-based grouping, similarities within a group might not always contemplate bioavailability, metabolism, or biological/mechanistic plausibility which can contribute to uncertainty in the similarity justification for “reading-across” (Schultz et al., 2015). Therefore, as mentioned in the fifth step, in addition to structural grouping based on EC regulation 1565/2000, an additional wide range of HCS *in vitro* studies is desirable for all flavor ingredients against the selected FGRs, in order to strengthen “read-across” assumptions and reduce uncertainty.

Once confirmed through *in silico* and additional *in vitro* and *in vivo* experimental data, the flavor toolbox approach has the potential to “predict” toxicity, reducing the time and resources needed to generate safety data on a large number of flavor ingredients, while minimizing the need for *in vivo* animal studies. The case study presented here demonstrates how this complex challenge could be addressed and provides a basis for future discussion and subsequent research of inhaled flavor ingredients.

## Data Availability

The original contributions presented in the study are included in the article/Supplementary Material, further inquiries can be directed to the corresponding author.
