# Supplementary material for: Toxicological Assessment of Flavor Ingredients in E-Vapor Products
Source: Front Toxicol. 2022 Apr 20;4:878976. doi: 10.3389/ftox.2022.878976 (PMC9065440; doi:10.3389/ftox.2022.878976)
Supplement: Supplementary file 2 [file Table1.docx]

**Toxicological Assessment of Flavor Ingredients in E-Vapor Products**

Davide Sciuscio, Florian Martin, Ashutosh Kumar, Britt Langston, Elyette Martin, Diego Marescotti, Carole Mathis, Julia Hoeng, Manuel C. Peitsch, Donna Smith, Maria Gogova, Patrick Vanscheeuwijck, K Monica Lee

**Online Resource 1**

**Conference references used in the framework manuscript**

Note: The scientific framework and early results were disclosed at scientific meetings as listed below.

1. D. Sciuscio1 , T. B. Langston2 , K. Ashutosh2 , D. C. Smith2 , D. Marescotti1 , F. Martin1 , K. M. Lee2 , J. Hoeng1 , and P. Vanscheeuwijck1 . 1 PMI R&D, Philip Morris Products S.A., Neuchâtel, Switzerland; and 2 Altria Client Services LLC, Richmond, VA.  Flavor Ingredients in E-vapor Products: A Structure-Based Grouping Approach to Predict Their Biological Activity. In 2021 *Annual Meeting*, Society of Toxicology, Abstract 2047, Poster board number P148
2. Sciuscio D, Ehman KD, Langston TB, Kumar A, Lee KM, Marescotti D, F. Martin, and P. Vanscheeuwijck. A Structure-Based Grouping Approach for Predicting Biological Activity of Flavor Ingredients Contained in E-vapor Products. In: 2020 *Annual Meeting Abstract Supplement*, Society of Toxicology, 2020. Abstract no. 3186
3. Marescotti D, McHugh D, Acali S, Nallet E, Huber A, Helbling F, Kabalan K, Leroy P, Biasoli M, Frauendorfer F, Sciuscio D, Utkarsh U, Lee KM, Peitsch M, Vanscheeuwijck P, and Hoeng J.  An Integrated *In Vitro* Mechanism of Action Assessment Approach for Evaluating E-cigarette Flavoring Compound Toxicity. In: 2020 *Annual Meeting Abstract Supplement*, Society of Toxicology, 2020. Abstract no. 3204
4. Wong E, Luettich K, Sciuscio D, Kumar A, Leroy P, Hoeng J, Lee KM, and Vanscheeuwijck P. A Structure-Based Grouping Approach to Evaluate Toxicity of e-Vapor Flavor Ingredients: Five-Week Inhalation Study in A/J Mice. In: 2020 *Annual Meeting Abstract Supplement*, Society of Toxicology, 2020. Abstract no. 3187
5. Smith CR et al. Preclinical testing of flavors in e-vapor products, part 2: Preparation and stability characterization of representative flavor mixtures. TSRC, Tob. Sci. Res. Conf., 2019, 73, abstract. 104
6. Zhang J. et al. Preclinical testing of flavors in e-vapor products, part 4: Flavor transfer from the liquid to the aerosol for inhalation exposure. TSRC, Tob. Sci. Res. Conf., 2019, 73, abstract. 106
7. Doshi U. et al. Preclinical testing of flavors in e-vapor products - Part 3: In vitro cytotoxicity and genotoxicity of representative flavor mixtures. TSRC, Tob. Sci. Res. Conf., 2019, 73, abstract. 105
8. Ehman K. et al. Preclinical testing of flavors in e-vapor products - Part 1: Selection of representative flavor mixtures for toxicological evaluations using a structural grouping approach. TSRC, Tob. Sci. Res. Conf., 2019, 73, abstract. 103
9. Glabasnia A et al. Importance of e-liquid preparation and stability characterization for a combinatorial safety assessment approach of flavors in e-liquids. CORESTA Meeting, Smoke Science/Product Technology, 2019, Hamburg, ST 13
10. Donna Smith, Davide Sciuscio, Cameron Smith, Patrick Vanscheeuwijck, Julia Hoeng. Beyond Science and Decisions: From Problem Formulation to Dose-Response Assessment, Workshop XI; February 18-20, 2020; Cincinnati, Ohio USA
