# Supplementary material for: Toxicological Assessment of Flavor Ingredients in E-Vapor Products
Source: Front Toxicol. 2022 Apr 20;4:878976. doi: 10.3389/ftox.2022.878976 (PMC9065440; doi:10.3389/ftox.2022.878976)
Supplement: Supplementary file 3 [file Table2.DOCX]

**Toxicological Assessment of Flavor Ingredients in E-Vapor Products**

Davide Sciuscio, Florian Martin, Ashutosh Kumar, Britt Langston, Elyette Martin, Diego Marescotti, Carole Mathis, Julia Hoeng, Manuel C. Peitsch, Donna Smith, Maria Gogova, Patrick Vanscheeuwijck, K Monica Lee

**Online Resource 2**

# **Structure determination**

The structures of 244 compounds were extracted from **SciFinder®** (https://scifinder.cas.org) by using the CAS number of each compound.

The compounds were imported into our internal chemical database, named **UCSD** (Unique Compound and Spectra Database; Martin E, Monge A, Duret JA, Gualandi F, Peitsch MC, Pospisil P. Building an R&D chemical registration system. *J Cheminform.* 2012 May 31;4(1):11. doi: 10.1186/1758-2946-4-11.). When compounds are imported into USCD, their structures are standardized (e.g., removal of salt, neutralization of the compound, and addition of hydrogens). Once they are registered in UCSD, the compounds are assigned a unique PMICODE.

For compounds that were present in the food flavoring database (**EFSA;** European Food Safety Authority), the following information were also extracted: Fl no., CE no. (Council of Europe number), JECFA No. (Joint FAO/WHO Expert Committee on Food Additives number), FGE.19 subgroup, and reference.

The ESFA database (https://webgate.ec.europa.eu/foods_system/main/?event=display) serves as an information tool on flavoring substances that have been approved for use in food in the European Union (EU) and included information on the conditions for use. It also contains flavoring substances that may currently be placed in the market until the risk assessment and authorization procedures are concluded. The information in this database is based on the Union list of flavorings and source materials as laid down in Part I of Annex I of Regulation (EC) No. 1334/2008.

# Predicted data

**QSAR ToolBox** (version 4.2; Schultz TW, Diderich R, Kuseva CD, Mekenyan OG. The OECD QSAR Toolbox Starts Its Second Decade. *Methods Mol Biol.* 2018;1800:55-77. doi: 10.1007/978-1-4939-7899-1_2.) was used to extract Cramer classes extended.

**Pipeline Pilot TOPKAT** module (BIOVIA, Dassault Systèmes, BIOVIA Pipeline Pilot, Release 2017; San Diego: Dassault Systèmes, [2018].) was used to predict:

- TOPKAT prediction rat inhalational_LC50_mg_m^3^_h

The rat inhalational LC50 (partial least squares) model predicts the median rat inhalational lethal concentration per hour (LC50) in an exposure toxicity test. This model was trained using 666 experimental rat inhalational LC50 values from open literature selected after critical review of experimental data. Only exposure times in the range of 0.5 to 14 h were accepted. Endpoints were modeled as -log10(C/hours_of_exposure).

In order to normalize the data for different durations of exposure, it was assumed that, within the range of adjustment, toxicity was proportional to duration. Thus, the units that were modeled were mole/m^3^/h. This normalization technique ignores the possibility that the slope at the observed time may not be the unit slope; but, as only a single time point was available, this information was lacking. The other possibility was to use the data only for identical exposure durations (for example, 2 or 4 h). But this alternative would severely limit the number of compounds in the training set; so, the former normalization method was used.

- TOPKAT_Ocular_Irritancy_None_vs_Irritant

The ocular irritancy (Bayesian) model predicts whether a particular compound is likely to be an ocular irritant. This model was trained using 1459 samples in the Draize test. The training set was selected from 1453 uniform studies after critical review of open literature. Modified Bayesian learning (described in Xia, Maliski, Gallant, and Rogers, J. Med. Chem., 47, 2004, pp. 4463-4470) was used to model the data.

- TOPKAT_Weight_of_Evidence_Rodent_Carcinogenicity#Prediction

The weight-of-evidence rodent carcinogenicity (Bayesian) model computes a true/false prediction by using the estimated best cutoff value. True indicates that a compound is a carcinogen. This discriminant model, derived from data provided by the US FDA CDER and from uniform studies selected after critical review of technical reports on rodent carcinogenicity studies conducted by NCI and NTP, computes the probability of a submitted chemical structure being a carcinogen. The model scores the chemical by using the FDA CDER weight-of-evidence protocol, which scores the chemical as a carcinogen if one of the following is true: 1. It is a multiple-site carcinogen in at least one sex/species combination (male or female/rat or mouse); 2. It is a single-site carcinogen in at least two sex/species combinations.

- TOPKAT_Chronic_LOAEL (mg/kg b.w.)

The rat chronic LOAEL (partial least squares) model predicts the chronic lowest-observed-adverse-effect level (LOAEL) of a chemical in rats. This model was trained by using 388 uniform experimental LOAEL values selected after critical review of open literature, NCI/NTP technical reports, and the US EPA databases.

- TOPKAT_Ames_Mutagenicity#Prediction

The Ames mutagenicity (Bayesian) model computes a true/false prediction by using the estimated best cutoff value. True means that the sample is a mutagen. This model was trained using 6313 samples from 6 different mutagenicity datasets:

1. TOPKAT version 6.2, Accelrys, San Diego, CA 92121
2. Kazius, McGuire, and Bursi, J. Med. Chem., 48, 2005, pp. 312-320
3. Contrera, J.F., Matthews, E.J., Kruhlak, N.L., and Benz, R.D., Regulatory Toxicology and Pharmacology 2005, pp. 313-323
4. Feng, J., Lurati, L., Ouyang, H., Robinson, T., Wang, Y., Yuan, S., and Young, S.S., J. Chem. Inf. Comput. Sci., 2003, pp. 1463-1470
5. Helma, C., Cramer, T., Kramer, S., and De Raedt, L., J. Chem. Inf. Comput. Sci., 2004, pp. 1402-1411
6. ISSCAN dataset, Istituto Superiore di Sanita (available at <http://www.epa.gov>).

Duplicates were removed across the datasets, and the compounds were assayed in accordance with the US EPA GeneTox protocol. According to the protocol, a chemical is tested by the histidine reversion assay against the following five strains of *Salmonella typhimurium*: TA100, TA1535, TA1537, TA 1538, and TA 98. Tests are performed both with and without S9 activation. A chemical is labeled a mutagen if a positive response (that is, a significant increase in the number of reversions relative to the background reversion) is observed with one or more strains, with or without S9 activation. Modified Bayesian learning (described in Xia, Maliski, Gallant, and Rogers, J. Med. Chem., 47, 2004, pp. 4463-4470) was used to model the data.

- TOPKAT_Developmental_Toxicity_Potential#Prediction

The developmental toxicity potential (Bayesian) model predicts whether a particular compound is likely to be toxic in a developmental toxicity potential assessment. True means toxic in this case. This model was trained using 270 samples in uniform experimental studies selected after critical review of approximately 3000 open literature citations. The data was modeled using Modified Bayesian learning (described in Xia, Maliski, Gallant, and Rogers, J. Med. Chem., 47, 2004, pp. 4463-4470).

# Literature data

If available, the following data were manually extracted from the **ECHA** (<https://echa.europa.eu>) registration dossier in February 2018 from the toxicological information part:

- Acute toxicity
  - Acute toxicity: oral (LD50)
  - Acute toxicity: inhalation (LC50)
- Repeated dose toxicity
  - Repeated dose toxicity: oral (NOAEL)
  - Repeated dose toxicity: inhalation (NOAEC)
- Genetic toxicity
  - Genetic toxicity: in vitro
    - In vitro gene mutation study in bacteria
    - In vitro gene mutation study in mammalian cells
    - Genetic toxicity in vitro
    - In vitro cytogenicity/chromosome aberration in mammalian cells
    - In vitro cytogenicity /micronucleus
    - In vitro DNA damage and/or repair study
  - Genetic toxicity: in vivo

Next to each extracted dataset, a column labelled R indicates the reliability linked to the endpoint.

The codes recorded in this column R indicate the following:

- K = key study
- S = supporting study
- W = weight of evidence
- O = other information
- 1 = reliable without restriction
- 2 = reliable with restrictions
- 3 = not reliable
- 4 = not assignable
- read-across = read-across based on grouping of substance or from supporting substance
- QSAR = data from a QSAR study

For genetic toxicity, only data from key studies (K) are reported.

For acute toxicity and repeated dose toxicity, supporting studies (S) are also reported in case no key study is available, and studies with weight of evidence (W) are reported if no key or supporting studies are available.

When there are several studies and only one reliability level is reported, this indicates that all studies have the same reliability level.
